# Innovations in Personalized and Targeted Therapies for Breast Cancer

**DOI:** 10.1155/2018/5409846

**Published:** 2018-11-14

**Authors:** Eleanor E. R. Harris, Jennifer De Los Santos, Virginia F. Borges, Stephen R. Grobmyer

**Affiliations:** ^1^University Hospitals Case Western Reserve University, 11100 Euclid Ave., Cleveland, OH 44106, USA; ^2^Grandview Cancer Center, 3670 Grandview Pkwy. Ste. 100, Birmingham, AL 35243, USA; ^3^Deputy Division Head, Medical Oncology, University of Colorado School of Medicine, Director, Breast Cancer Research Program, 13001 E. 17th Pl., Aurora, CO 80045, USA; ^4^Lerner College of Medicine, Cleveland Clinic, 9500 Euclid Avenue/A81, Cleveland, Ohio 44195, USA

This special issue highlights many of the innovations and diverse methodologies for personalizing the treatment of breast cancer using novel assays and therapies targeting the molecular mechanisms driving individual tumor biology. In the current era of precision medicine, breast cancer treatment is a leading model for truly individualized diagnosis and treatment, as highlighted in our issue's reviews and original articles. This innovative approach to cancer therapy is occurring across the array of diagnostic and treatment modalities as our authors' contributions demonstrate. In particular this issue seeks to highlight the advances in precision medicine as it applies to the surgical and radiotherapy approaches to more individualized surgical and radiation treatments.

The quest to characterize breast cancers by molecular subtype has been pursued for decades, since the advent of estrogen and progesterone receptor expression as the simplest manifestation of subtyping. Molecular characterizations predict tumor behavior and prognosis and provide potential targets for systemic therapy and novel drug development. The breast develops under the influence of multiple endocrine pathways, and there are many endocrine therapies targeted to hormone receptor expressing breast cancers, with avid research ongoing to further define these molecular targets. Recent work on both androgen receptor and glucocorticoid receptor expression in the differentiation and tumorigenesis pathways of the breast provide potential new molecular targets for systemic therapies. At the other end of the biological spectrum are those breast cancers devoid of any established hormone receptors, the triple negative cancers, which are a heterogeneous group with a poorer prognosis and the subject of intense study to identify more effective systemic agents and delivery methods. One such approach is the use of continuous chemotherapy dosing, called metronomic delivery, designed to avoid any break in the systemic therapy that could allow tumor cell proliferation. Other investigations are looking at intensification of chemotherapy, new agents, and biologics including PARP and other DNA-damage repair inhibitors and immunotherapy drugs. Metastatic breast cancer is increasingly viewed as a chronic disease that can be managed and controlled rather than a terminal illness. An active research topic is the integration of radiotherapy to release tumor antigens and immunotherapy in the treatment of oligometastatic disease, with the goal of long term survival or even cure.

Local regional treatment of operable breast cancer over recent decades has evolved to embrace a “less is more” philosophy, from the adoption of breast conserving therapy, to the validation of sentinel node biopsy and more recently investigations into omission of surgery or radiation for biologically low risk cancers. The goal is to optimize the treatment for the individual patient based on the predicted capacity of their breast cancer to recur locally, regionally, or systemically as predicted by multiple molecular markers and predictive gene panels. Without such information, patients are treated according to broad protocols and often receive more, or less, therapy than is indicated. One very innovative and promising area of active clinical research is the development of gene assays to predict tumor radiosensitivity. If an individual tumor can be tested for its response to radiation, the radiation dose and target volumes for treatment can be truly individualized to ensure optimal tumor control while minimizing toxicity and cost.

Patient centered value-based medicine is increasingly embraced as is patient preference and shared decision making. Patients are increasingly choosing more individualized approaches that meet their needs for not only treatment outcomes, but also quality of life, functionality, cost, and impact on their lives beyond the clinic. Clinicians are increasingly evolving from rigid adherence “cookie cutter” protocols to personalized treatment incorporating a vast array of integrated therapies. Many patients are opting for less extensive surgery, shorter courses of radiation, and optimized systemic therapies directed by predictive gene assays. Clinical trials are actively assessing ways to “right size” therapy, including omission of surgical, radiotherapy, and systemic treatments that are not therapeutic as well as abbreviating and accelerating radiotherapy regimens to improve efficacy and adherence.

Precision medicine in oncology seeks to individualize each patient's treatment regimen based on an accurate assessment of the risk of recurrence or progression of that person's cancer. Precision can be achieved at each phase of care, including detection, diagnosis, surgery, systemic therapy and radiation therapy, survivorship, and follow-up care. The precision arises from detailed knowledge of the inherent biological propensities of each tumor, rather than generalizing treatment approaches based on phenotypic or even genotypic categories. Extensive research is being conducted in multiple disciplines, including radiology, pathology, molecular biology, and surgical, medical, and radiation oncology. Clinical trial design is adapting to the new paradigms and moving away from grouping heterogenous patient populations into limited treatment comparison arms.

